# Relatively Benign yet a Reversible Cause of Dilated Cardiomyopathy

**DOI:** 10.1016/j.jaccas.2021.04.004

**Published:** 2021-06-02

**Authors:** Faris Khan, Mansoor Ahmad, Sumera Kanwal, Jason Payne, Shane Tsai, Daniel Anderson

**Affiliations:** Department of Internal Medicine, Division of Cardiovascular Medicine, University of Nebraska Medical Center, Omaha, Nebraska, USA

**Keywords:** atrial premature contractions, dilated cardiomyopathy, APC, atrial premature contraction, CS, coronary sinus, LVIDd, left ventricular internal diameter at end-diastole, LVIDs, left ventricular internal diameter at end-systole

## Abstract

Arrhythmia-induced cardiomyopathy secondary to frequent ventricular premature contractions is a well-studied phenomenon; however, there is a paucity of data showing a similar association with frequent atrial premature contractions (APCs). Early recognition and successful APC ablation can reverse left ventricular dysfunction in these patients. (**Level of Difficulty: Beginner.**)

## Central Illustration

**Figure:**
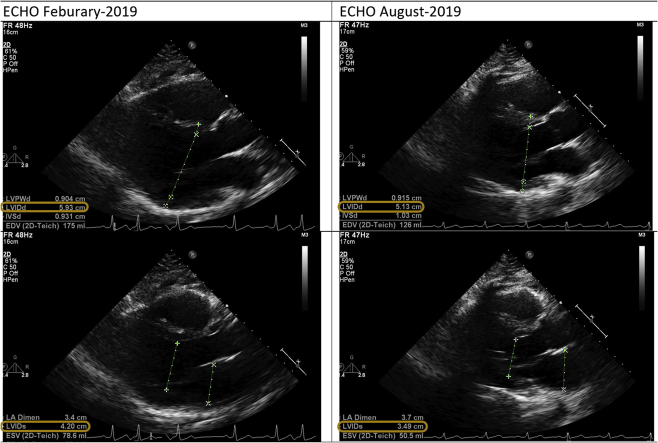

## Introduction

A high burden of ventricular premature contractions leading to reversible cardiomyopathy (1) has been well reported. In contrast, there are limited data showing an association of atrial premature contractions (APCs) with cardiomyopathy. This report presents the case of a young patient with a long-standing history of APCs leading to left ventricular dysfunction that resolved after a catheter-based ablation procedure.

**Learning Objectives**

• To be able to identify a high burden of APCs as a possible cause of reversible cardiomyopathy even in asymptomatic patients.
• To be able to use echocardiographic parameters such as LVID to determine the timing of an ablation in patients with frequent asymptomatic APCs.

## History of Present Illness

A 28-year-old man was referred to the arrhythmia clinic at the University of Nebraska Medical Center in Omaha, Nebraska with an initial diagnosis of frequent APCs since childhood and left ventricular dysfunction. He did not have any significant symptoms except occasional palpitations.

## Past Medical History

He had history of obesity and frequent APCs; however, he did not report obstructive sleep apnea or excessive intake of caffeinated beverages. His family history was unremarkable. On physical examination, he was normotensive, with a body mass index of 39.8 kg/m^2^ (weight, 262 lbs; height, 1.727 m). The rest of the examination was unremarkable.

## Differential Diagnosis

The differential diagnosis of left ventricular dysfunction included tachycardia-induced cardiomyopathy secondary to frequent APCs versus primary dilated cardiomyopathy.

## Investigations

**Figure 1:**
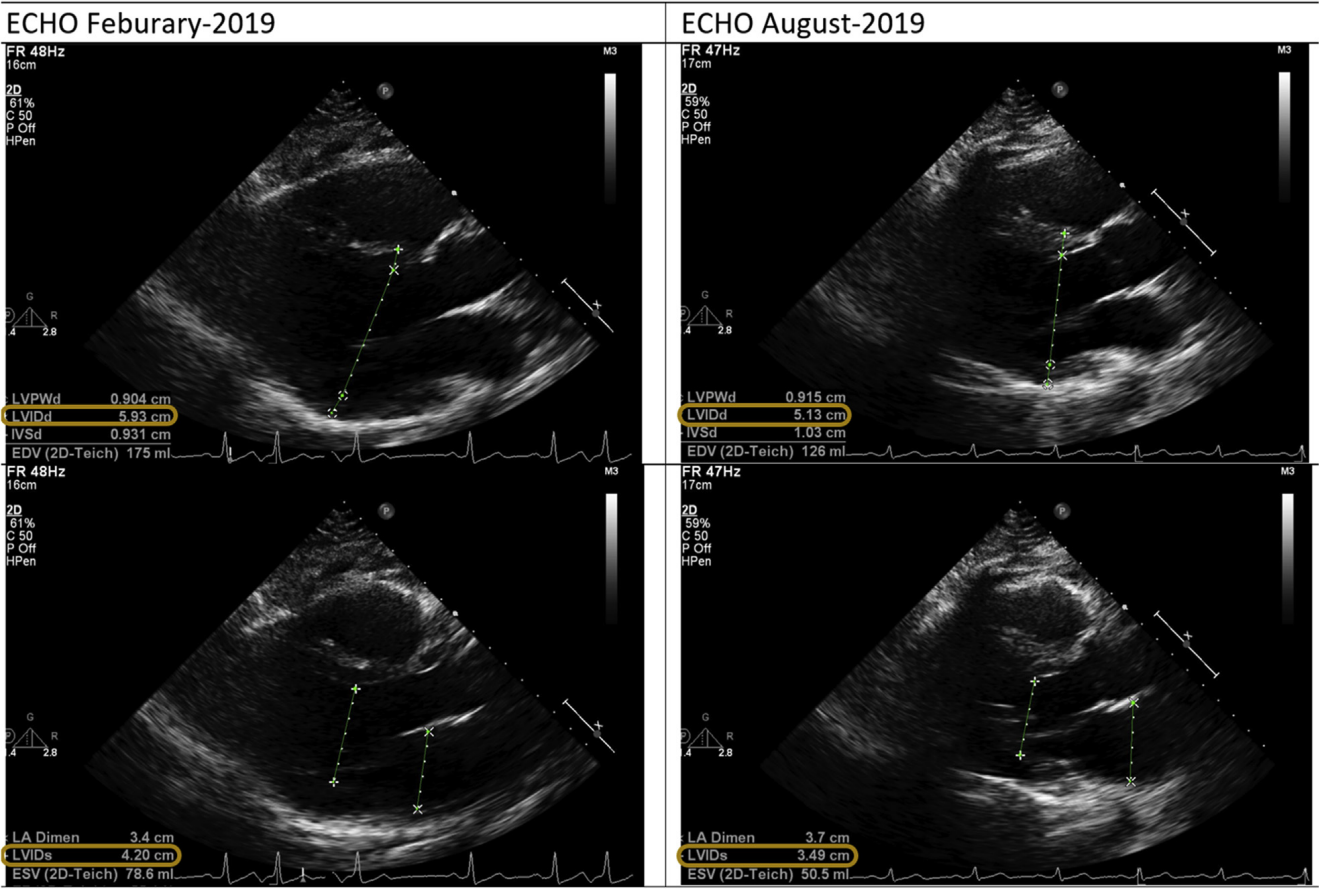
Echocardiographic Parameters, Including LVIDd and LVIDs, Before and After APC Ablation APC = atrial premature contraction; C = contrast; ECHO = echocardiogram; EDV = end-diastolic volume; ESV = end-systolic volume; FR = frame rate; IVSd = inter-ventricular septum during end-diastole; LA Dimen = LA dimension; LVIDd = left ventricular internal diameter during end-diastole; LVIDs = left ventricular internal diameter during end-systole; LVPWd = left ventricular posterior wall during end-diastole; HPen = harmonic pen; P = persistence; 2D = 2-dimensional.

His 48-h Holter monitor in February 2019 showed an excessive APC burden of 37% (average heart rate 92 beats/min [range 56 to 161 beats/min]). His previous 24-h Holter monitor in September 2015 showed a 36% APC burden (average heart rate 87 beats/min [range 54 to 171 beats/min]). He had an echocardiogram in February 2019 that showed a dilated left ventricle (left ventricular internal diameter [LVID] at end-diastole [LVIDd] and at end-systole [LVIDs] were 5.9 and 4.2 cm, respectively) (Figure 1), with an ejection fraction of 35% to 40%. There was corresponding atrial ectopy with a burden of nearly 50% at the time of the study. Right ventricular function was normal. No significant valvular abnormality was detected.

**Figure 2:**
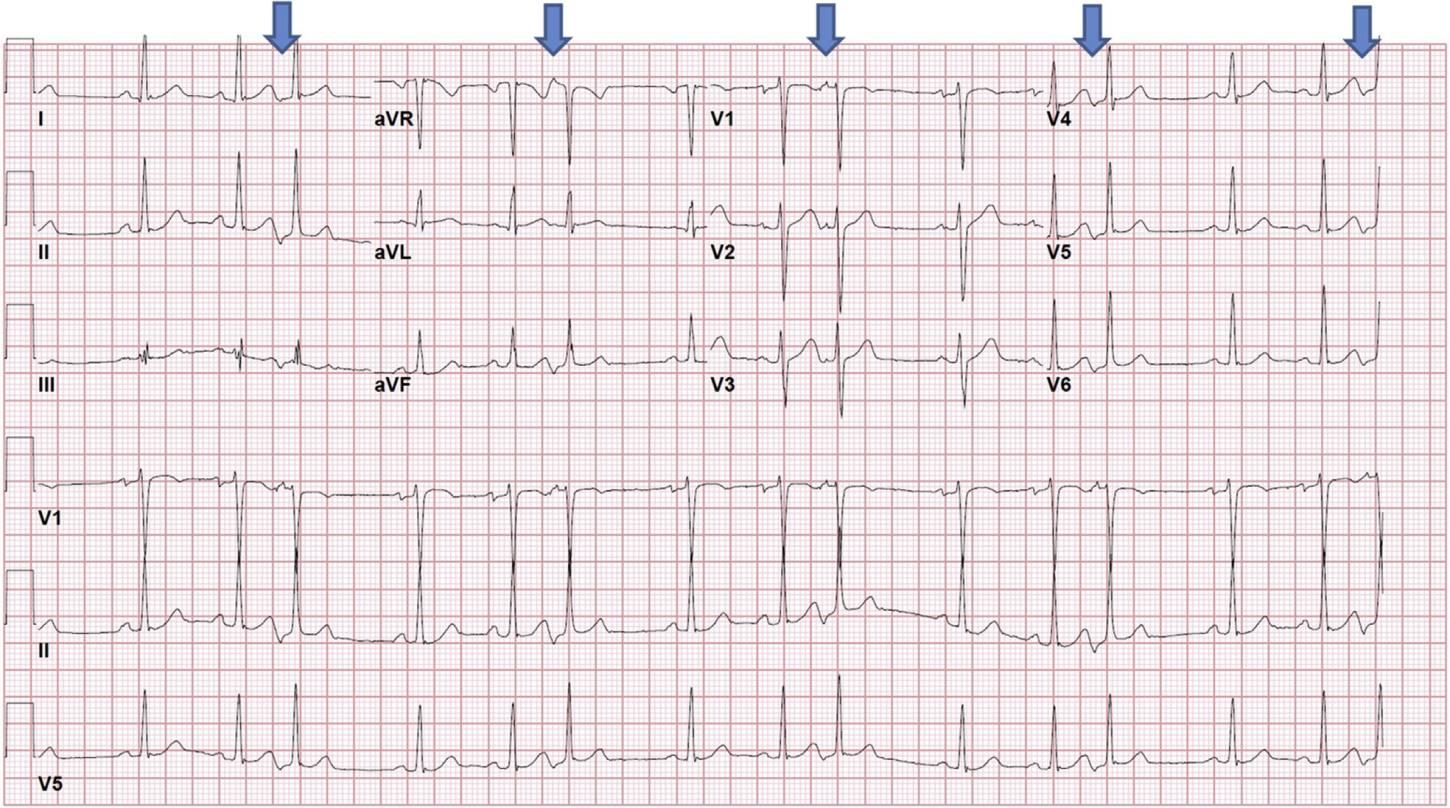
Electrocardiogram Showing Atrial Premature Contractions in Trigeminy **Arrows** point to the arial premature contractions.

The vector analysis showed the following APC wave configuration on his 12-lead electrocardiogram (Figure 2): a negative P-wave in leads II, III, and AVF; a positive P-wave in leads AVR and AVL; a positive P-wave in leads V_1_ to V_3_; a negative P-wave in leads V_3_ to V_6_; and isoelectric in lead I. On the basis of the configuration, the APC appeared to be originating from the coronary sinus (CS) body on vector electrocardiography.

## Management

**Figure 3:**
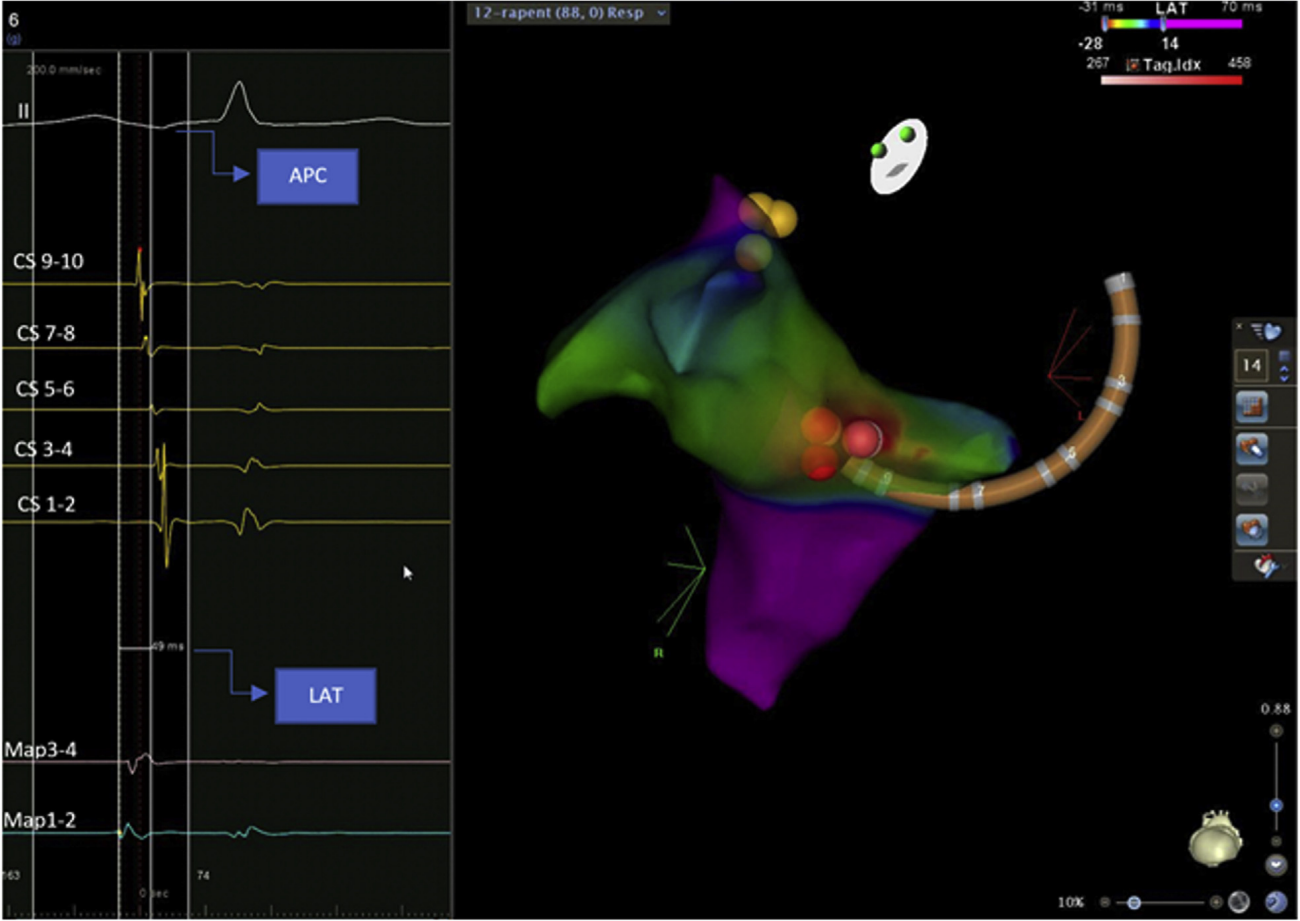
Local Activation Timing Map With Ablation Lesions The **red** CARTO VISITAG (Biosense Webster, Irvine, California) represents ablation lesions in a left anterior oblique caudal view; **yellow dots** represent the His bundle. APC = atrial premature contraction; CS = coronary sinus; LAT = local activation timing.

He was previously prescribed metoprolol; however, it was discontinued because of intolerance. After shared decision making, the patient elected to proceed with an electrophysiology study in May 2019 to rule out tachycardia-induced cardiomyopathy secondary to frequent APCs. On mapping, the earliest activation timing site was initially localized to the left atrial floor near the CS os (local activation time was 41 ms earlier than surface P-wave). However, after application of a few ablation lesions using a Thermocool SmartTouch SF contact force-guided radiofrequency ablation catheter (Biosense Webster, Irvine, California) (maximum power 35 W with a target ablation index of 400), the left atrium was no longer the earliest site, and the change in configuration was consistent with a shift to the right atrium. Additional mapping was done in the right atrium as well as in the CS. The earliest activation timing site was ultimately localized near the CS os (local activation time was 49 ms earlier than the surface P-wave) (Figure 3). After bracketing the area, ablation lesions were applied (maximum power 35 W with a target ablation index of 400), with cessation of APCs. There was no recurrence of APCs even after isoproterenol infusion.

## Discussion

APCs are usually considered benign; however, if APCs occur in high frequency, they can potentially lead to dilated cardiomyopathy. We present the case of a young patient with frequent APCs leading to cardiomyopathy that reversed after successful radiofrequency ablation and elimination of the APCs. The pathophysiology of APC-induced cardiomyopathy is not well understood. In an animal study by Pacchia et al. (2), atrial pacing in a bigeminal fashion led to cardiomyopathy in dogs as a result of prematurity of ectopic beats but independent of dyssynchrony. Neurohormonal activation may also be involved in APC-induced cardiomyopathy. APCs can also cause myocyte remodeling, thus affecting heart function (3). It is also possible that atrioventricular dyssynchrony resulting from frequent APCs can cause beat-to-beat volume shifts with resultant stress and inflammation of myocytes leading to cardiac remodeling and dysfunction. Unlike ventricular premature contraction–induced cardiomyopathy (4), there is neither a clear cutoff of APC burden (5) nor a clear association of APC coupling interval with potential cardiomyopathy. Mazzella et al. (6) reported 19.9%, Liuba et al. (7) reported 19%, and Hasdemir et al. (8) reported 20.9% APC burden associated with cardiomyopathy that resolved after catheter-based ablation procedures. In our patient, the APC burden was more than 35%, with a coupling interval of 465 to 495 ms on the 12-lead electrocardiogram (400 to 410 ms on intracardiac electrocardiograms) that led to cardiomyopathy. A retrospective study by Gunda et al. (9), however, showed that frequent APCs are not associated with cardiomyopathy. However, the results of the report by Gunda et al. (9) cannot be generalized because they included only those patients who had an echocardiogram performed within 6 months (before or after) of a 14-day cardiac monitor. Conversely, APC-induced cardiomyopathy is usually a long-term phenomenon, as evidenced by our case, as well as other reports (6,7). Another reason for the statistically nonsignificant findings of APC-induced cardiomyopathy by Gunda et al. (9) is that only 62 patients had an APC burden >5% in a total study group of 846 patients. This is a very small proportion of the whole study group and is not sufficient to study such a rare phenomenon. Moreover, the APC burden cutoff of more than 5% provides a wide range that would affect the outcomes of the study. The same study also reported negative results for cardiomyopathy in 7 experiments in animals exposed to APCs for 12 weeks. Again, the short duration of APC exposure may not have been sufficient to induce cardiomyopathy. Additional studies with large sample sizes and longer follow-up durations are required to investigate this phenomenon.

## Follow-Up After Ablation

The patient had a follow-up 48-h Holter monitor in July 2019 (without any medical therapy) that showed sinus rhythm with only 1 isolated APC (average heart rate 81 beats/min [range 48 to 144 beats/min]). A follow-up echocardiogram in August 2019 showed that right ventricular systolic function was normal and left ventricular function had recovered (ejection fraction was 50% to 55%; LVIDd and LVIDs were 5.1 and 3.5 cm, respectively). APCs were not evident at the time of the study. Vervueren et al. (5) reported similar improvement of LVIDd from 7.1 to 5.8 cm after successful APC ablation.

## Conclusions

APC-induced cardiomyopathy can occur in patients with a high APC burden. Therefore, we recommend regular monitoring of cardiac function with an echocardiogram even in asymptomatic patients. If left ventricular dilation or dysfunction is noticed, catheter-based ablation therapy can offer excellent results.

## Funding Support and Author Disclosures

The authors have reported that they have no relationships relevant to the contents of this paper to disclose.
